# The Effects of Training in Music and Phonological Skills on Phonological Awareness in 4- to 6-Year-Old Children of Immigrant Families

**DOI:** 10.3389/fpsyg.2016.01647

**Published:** 2016-10-21

**Authors:** Hanne Patscheke, Franziska Degé, Gudrun Schwarzer

**Affiliations:** Department of Developmental Psychology, Justus-Liebig-UniversityGiessen, Germany

**Keywords:** preschoolers, children of immigrant families, language, music training program, phonological awareness

## Abstract

Children of immigrant families often have great difficulties with language and disadvantages in schooling. Phonological problems appear especially common. Thus, the aim of this study was to determine whether music training has a positive effect on the phonological awareness in these children. The effects of a music program were compared with an established phonological skills program and with a sports control group. Preschoolers of immigrants (19 boys, 20 girls) were randomly assigned to one of the three groups. All groups were trained three times a week for 20 min each, over a period of 14 weeks. Phonological awareness was tested prior to the beginning of the training and after the training phase. At the pre-test, no differences between the groups were found regarding phonological awareness and control variables (age, gender, intelligence, socioeconomic status, language background, music experience). At the post-test, the music group and the phonological skills group showed a significant increase in phonological awareness of large phonological units. The effect size of the music training was larger compared to the phonological skills program. In contrast, the sports control group showed no significant increase in phonological awareness. The current results indicate that a music program could be used as an additional opportunity to promote phonological skills in children of immigrant families.

## Introduction

Empirical evidence indicates that phonological awareness is an important and reliable predictor of later reading and writing ability (Bus and van Ijzendoorn, 1999; Hartmann and Kessler, 2002; Küspert and Schneider, 2003; Marx et al., 2005). Phonological awareness is the individual sensitivity to the sound structure of language and describes the ability to analyze and manipulate language on the word level (large phonological units) and on the phoneme level (small phonological units). Especially for children of immigrant families, the acquisition of phonological awareness of a second language is an enormous challenge (Baumert and Schümer, 2001; Triarchi-Herrmann, 2006; Marx, 2007). Frequently, this leads to language difficulties that, in turn, can lead to school problems (Schwippert and Schnabel, 2000; Schwippert et al., 2003; Grimm and Aktas, 2004). Several well-established programs do exist to enhance phonological awareness (e.g., Küspert and Schneider, 2003). However, in addition to these programs, music training could also support the increase of phonological skills. Studies have discovered a positive effect of music training on phonological awareness (e.g., Gromko, 2005; Degé and Schwarzer, 2011). The influence of music training on phonological awareness might complement already existing language programs, because music is motivating and trains language without focusing on language deficits. Therefore, children of immigrant families who are less familiar with the ambient language might especially benefit from a music training program. However, it remains unclear whether music training could enhance the phonological awareness of children of immigrants. Thus, the aim of this study was to determine whether music training has a positive effect on the phonological awareness in these children.

### Phonological Awareness

Phonological awareness describes the understanding, detection, and manipulation of a language sound system on two levels: (1) On the word level, the ability refers to larger phonological units and comprises the isolation of individual words from the speech flow, blending and segmentation of chunks within words, and rhyming of words. (2) On the phoneme level, the ability refers to smaller phonological units and describes the manipulation of individual sound units (phonemes) within words. The development of phonological awareness is an implicit process until children learn the alphabet. With knowledge of letters, phonological awareness increases, because phonemes become audiovisual and explicit processes (Marx, 2007). Typically, children begin by segmenting spoken sentences into words. Then, they are able to segment words into syllables, and finally, they segment words into individual phonemes. Hence, the acquisition and advancement of phonological awareness is an important basis for reading and writing processes, especially for preschoolers before starting school (Marx, 2007). For this reason, researchers have developed well-established language programs that target phonological awareness (Küspert and Schneider, 2003; Plume and Schneider, 2004). In fact, many studies provide evidence that language programs enhance phonological awareness (Holdaway, 1979; Lundberg et al., 1988; Bus and van Ijzendoorn, 1999; Schneider et al., 1999, 2000; Stuart, 1999, 2004; Weber et al., 2007). Recently, the potential influence of music training on phonological awareness was also investigated because of the evidence for the association between music and language (e.g., Douglas and Willatts, 1994; Cutietta, 1995, 1996; de Frence, 1995; Lowe, 1995; Butzlaff, 2000; Fisher, 2001; Moreno et al., 2009).

### Music Training and Phonological Awareness

From the beginning of life, music and language are connected with each other; both consist of auditory stimuli, are generically structured, and deliver messages (Lehrdahl and Jackendoff, 1983). In addition, music and language share neural resources and use similar information processing (Koelsch and Siebel, 2005; Schön et al., 2010; Patel, 2011, 2014).

The literature provides different models that try to explain how musical training can enhance language abilities. Patel (2008) postulates that music and language share the same mechanism for learning sound categories (“shared sound category learning mechanism hypothesis”; SSCLMH). Accordingly, the categorical building blocks of music - musical notes - are related to the categorical building blocks of language - phonemes. The OPERA hypothesis from Patel (2011) can be seen as a global model that sheds extensive light on the domains of music and language, in order to explain how music training might impact on speech processing. “OPERA” stands for five conditions that are necessary for producing transfer effects: (1) Overlap; training has to tap into a common neural circuit for music and speech, (2) Precision; high precision is needed in order to trigger top-down tuning from cortical to subcortical sound encoding structures, (3) Emotion; emotion plays an important role because music offers emotional rewards, (4) Repetition; an indispensable learning principle which is necessary for plasticity to occur, and (5) Attention; refers to the importance of engaging focused attention while training.

Another approach assumes an increased sensitivity to speech sounds (Besson et al., 2011). Besson et al. (2011) justify their hypothesis with the functional overlap of brain structure in music and language processes and with findings of musicians’ increased sensitivity to acoustic parameters that are similar for music and speech. Derived from musical training, musicians have an enhanced sensitivity to auditory parameters that are important for music, such as frequency and duration. These parameters are involved in music and speech processes. Thus, the increased sensitivity in music perception leads to an elaborated perception of speech. That, in turn, facilitates speech processing. Recent studies provide evidence for an increased sensitivity to subtle speech cues facilitated by music training such as voice onset time (VOT) and speech segmentation (Chobert et al., 2014).

Related to the acoustical sensitivity assumption, Tierney and Kraus (2014) postulate the precise auditory timing hypothesis (PATH). They assume that music and language rely on extremely subtle timing details in sounds and that entrainment practice is the core mechanism of both. Music training requires entrainment, which, in turn, calls for precise perception of acoustic event timing. Thus, music training might promote timing precision that facilitates speech sound perception, which is important for phonological skills. Taken together, auditory features promoted by music training might have positive effects on language, particularly on phonological awareness.

Therefore, a number of studies have investigated the relationship between music and phonological awareness. Indeed, relationships between musical abilities (e.g., pitch perception) and phonological awareness have been identified (Lamb and Gregory, 1993; Anvari et al., 2002). Accordingly, researchers have investigated the association between music and language with respect to the effects of music training on phonological skills (e.g., Gromko, 2005; Bolduc, 2009; François et al., 2013; Moritz et al., 2013). Moritz et al. (2013) investigated whether kindergartners who received 45 min of musical training every day demonstrated more phonological skills than kindergartners who received 35 min of music training per week. Both groups were trained for a period of 1 year. After the training phase, children who received more music training showed improvements in a wider range of phonological awareness skills. However, due to a lack of control groups and content differences between the music programs, the training effects cannot be interpreted unequivocally. Bolduc (2009) trained 5-year-old children in kindergarten with two different music programs, but both were comparable regarding the procedure (time, frequency). One was designed to increase reading and writing, and the other to enhance musical abilities. Results showed that the music program that addressed reading and writing was more efficient in promoting phonological awareness than the second music program. Unfortunately, Bolduc had no control group without music in his study, so it remains unclear whether both programs enhanced phonological awareness, or the effects occurred simply due to maturation and a Hawthorne effect. In a study from Gromko (2005), kindergarten children were trained with a music program for 4 months. The study revealed a significant increase in phonological awareness, particularly in phoneme segmentation fluency. However, due to a pseudo-random assignment of the children to the treatment or no-treatment control group, it is not possible to interpret the results with complete certainty. It is also possible that the treatment group represented an effect of extra attention compared to the no-treatment control group. To address these concerns, Degé and Schwarzer (2011) conducted a study and tested the effect of a music program on phonological awareness by randomly assigning 5- to 6-year-old preschoolers to a music program, a phonological skills program, or a sports control group. The preschoolers were trained in small groups for 10 min every day for a period of 20 weeks. Phonological awareness was assessed before and after the training phase. The results indicated that the music program had a positive effect on phonological awareness in 5- to 6-year-old preschoolers. With respect to phonological awareness of large phonological units, the effect size of the music program (*dcorr* = 0.9) was comparable to that of the phonological skills program (*dcorr* = 0.6) that directly addressed phonological awareness. Thus, this training study, with trained experimental and control groups, could clearly show that music can train phonological awareness. A similar conclusion was reached from a recently published meta-analysis by Gordon et al. (2015) that dealt with the issue of whether music training studies enhance literacy skills, including phonological awareness. They included 13 studies (out of 901) that met the criteria that included control groups, a pre-post measure, and reading instruction that was held constant across groups. Their result supports the hypothesis that music training promotes phonological skills.

Taken together, these studies present the highly interesting result that music trains phonological awareness without focusing on language deficits. These findings may prove useful for groups of children with deficits in language acquisition.

### Children of Immigrant Families and Language Acquisition

Prior studies demonstrate that children of immigrants often have great difficulties with language because they learn a second language only outside of their home environment (Baumert and Schümer, 2001; Triarchi-Herrmann, 2006; Marx, 2007). Phonological problems appear especially often (Triarchi-Herrmann, 2009). Frequently, these language difficulties lead to school problems, where poor academic performance cannot be attributed to intelligence but to an enormous deficit in the children’s language abilities (Schwippert and Schnabel, 2000; Schwippert et al., 2003; Grimm and Aktas, 2004). Possible reasons for these serious language problems are a missing language model for the grammatical structure of the second language and the rare opportunity to practice the second language in the home environment (Tracy, 2009). Furthermore, Baumert and Schümer (2001) indicate that an inadequate governmental integrative structure for immigrants is also responsible for the language problems. The study revealed large differences in school performance between children of immigrant families and children without such a background.

Although there exist established language programs that address language problems and enhance phonological awareness (as the previously cited literature presents), there are still many children of immigrants that have reading and writing difficulties and, therefore, disadvantages in schooling (Marx, 2007). Hence, it might be helpful to support their language development with additional training opportunities such as through musical training. A study from Herrera et al. (2011) investigated the effects of phonological and musical training on phonological awareness in native- and foreign-Spanish speaking children aged four. The 2 year pre-test/post-test study assigned 97 preschoolers to three groups using a stratified randomization procedure: One experimental group received phonological training based on isolated words, a second experimental group received phonological training that was based on children’s rhymes and songs, a third group acted as a control group receiving no specialized training. All groups had about the same number of native- and foreign-Spanish speaking children. The two experimental groups were identical regarding organization (e.g., scheduling), materials (e.g., pictures), and content (e.g., words). The experimental groups outperformed the control group in the post-tests in all measured phonological awareness tasks (e.g., rhyming, segmenting, phoneme detecting) regardless of the native language of the children. However, Herrera et al. (2011) did not use a comprehensive music training program; rather, they modified the impartment of an existing phonological training program by embedding the isolated words from the phonological training into short songs. So it remains unclear how far the music itself was responsible for producing the effects in phonological awareness. A finding by Kilgour et al. (2000) demonstrated that the recall of spoken and sung lyrics is mediated by the presentation rate, rather than by the inclusion of melody. When the presentation rate was manipulated so that the durations of the spoken and the sung materials were equal, there was no advantage for sung over spoken lyrics, as it was the case when there was no manipulation. In other words, songs could have been a memory aid that in turn might have contributed to the results by Herrera et al. (2011). A comprehensive music training program containing all aspects of music like rhythmic exercises, meter execution, joint singing, pitch perception, and intonation might be more motivating than a typical phonological program because it does not focus on language. An exclusive focus on language could lead to motivational problems for children of immigrants because they are confronted with their weaknesses.

### Objectives

Investigating the effect of a music program on phonological awareness in children of immigrant families is desirable and necessary as little research on this topic has thus far been conducted. This target group needs particular help due to their serious deficits in language acquisition and reading and writing abilities. The indirect method of enhancing their phonological awareness with a comprehensive music training program could be a motivating and enriching alternative or complement to a direct phonological skills program. Previously, Hesse (2003) emphasized the engaging quality of musical activity. Wolf et al. (2000) highlight the fact that engagement facilitates learning; thus, children of immigrants could benefit from music by improving their language skills (Moritz et al., 2013).

To this end, our study investigated the effect of a music program on phonological awareness in children of immigrants using the same method as Degé and Schwarzer (2011). The effects of a music program were compared with an established phonological skills program that explicitly addresses phonological awareness. The effects of a music program were also compared with a control group that received sports training, to control for the effects of retesting, maturation, and attention. In this experiment, we assigned 4- to 6-year-old preschoolers of immigrant families to one of the three groups to control for systematic differences between the groups. All groups were trained three times a week for 20 min each over a period of 14 weeks. Phonological awareness was tested prior to the beginning of the training and after the training phase. Thus, it was possible to establish a specific causal relationship between music training and phonological awareness in our experimental groups.

## Materials and Methods

### Participants

The study was conducted in full accordance with the Ethical Guidelines of the German Association of Psychologists (DGPs). In accordance with the ethical guidelines mentioned above informed consent was obtained from the parents for each participant. Participants were recruited from kindergartens in Germany. The sample consisted only of children of immigrant families. The criterion for the migration background was that either the children or at least one of their parents were born in a foreign country. At the beginning of the experiment, the sample comprised 62 preschoolers (30 boys; 32 girls) with an age range from 4.8 to 6.9 years, (*M* = 5.11 years, *SD* = 5.4 month). Only participants with at least 70% training attendance were included in the final analyses. Overall, the remaining participants numbered 39 preschoolers (19 boys, 20 girls), with an age range from 4.8 to 6.8 years, (*M* = 5.11 years; *SD* = 5.3 month).

Only 18% of the children had prior music training experience (e.g., taking private music lessons, singing in a choir, etc.) as reported by parents (see “Materials – Measures” questionnaire of sociodemographic data). Furthermore, 82% of the parents reported having no music training experience. Socioeconomic status was measured by parents’ education levels and monthly income. The educational background information revealed that for 62% of the children, neither parent had a university degree; for 8%, one parent had a university degree; and for 7%, both parents had a university degree. The parents of the remaining 23% did not provide details about their education. The monthly income range was between 1000 Euro and 3000 Euro, with 39% of the parents reporting an income between 1000 Euro and 2000 Euro, 17% reporting between 2000 Euro and 3000 Euro, and 44% of the parents providing no details about their monthly income. The ethnic origin of most of the families was Kurdish (69%); 23% were Russian, 5% were Asian, and 3% were American. Germany was the country of birth for 90% of the children; however, most of the parents were born in a foreign country (92% men, 77% women). Almost all families (90%) had lived in Germany for more than 5 years. Many families reported speaking little German at home – an indication of a poor German language level (Tracy, 2009); **Table 1** for more details. A third of the children (33%) attended a special preschool program provided by schools and kindergartens to prepare children who failed the school enrollment examination for starting school. In this program, the children go to school twice a week for 2 h per session and acquire basic knowledge that is presupposed for preschoolers in that age group (e.g., naming of colors, numbers, and geometrical shapes, animals, traffic issues). The participants were randomly assigned to a music program, a phonological skills program, and a sports control group.

**Table 1:** Reported languages spoken at home by the immigrant families.

Language	%
Only native language, no German	18
Mostly native language, hardly any German	41
Equally native language and German	18
Mostly German	20
Only German	3

### Material

#### Training Programs

Children were trained for 20 min three times a week for a period of 14 weeks. Trained research assistants implemented all three programs (music, phonological skills, sports). Research assistants met weekly to go over the manual based programs in order to ensure treatment fidelity. This process was supervised setting high value on compliance regarding correct order and execution of tasks as well as continuous reflection of the training sessions of all three groups. Preschoolers were trained at the kindergartens in a separate and undisturbed room. A typical session comprised a short welcome (small talk, attendance check) and three to four tasks of approximately 15 min.

The music program was arranged by Degé and Schwarzer (2011). It was based on a well-established program for early music education (Nykrin et al., 2007). The manual contained joint singing, joint drumming, rhythmic exercises, meter execution, training of rudimentary notation skills, dancing, and playful familiarization with intervals. Typical sessions comprised, for example, the learning of a new song, a meter execution, and a listening exercise. First, all children listened to the song. Then they attempted to sing along with the trainer. Finally, the group sang the song without the trainer. The second task comprised dancing to certain musical themes or synchronization of particular body parts to the music, to feel the rhythm and the meter with the whole body. The third task involved listening to music recordings and subsequently identifying the tempo or musical instruments in the recording. Other typical sessions involved familiarization with different instruments and joint drumming. The children had the opportunity to test several instruments by following the instructions of the trainer. Joint drumming activities involved synchronization with a given beat by the trainer as well as the creation of new simple beats by the children that had to be learned by the other group members. When the participants had an advanced knowledge of the different instruments and songs, the tasks became more complex, such as joint singing of familiar songs, and drumming while singing.

The phonological skills program was a well-established program specifically designed to train phonological awareness (Küspert and Schneider, 2003). The manual contained listening exercises, rhyming exercises, phoneme recognition exercises, syllable exercises, and the introduction of the concepts of “word” and “sentence.” During the first weeks of training, typical sessions contained listening tasks that involved every day sounds and rhyming with animal names. Children closed their eyes and guessed the sounds that the trainer produced, or chose words that rhymed with an animal name such as “cat.” Other commonly used tasks were clapping the syllables of the animal, plant, or object displayed on a picture card as well as guessing the last phoneme of a word given by the trainer. Typical sessions at the end of the training phase included more difficult tasks such as the identification of words in sentences or the identification of onset phonemes of words.

The sports training was compiled by Degé and Schwarzer (2011) and the manual contained exercises to support motor skills and body coordination by training balance, physical strength, endurance, body perception, and relaxation. It was based on *Yoga and Active Games for Kids* from Dunemann-Gulde (2005). For example, typical sports sessions contained relaxation exercises and well-directed throwing and balancing tasks. For relaxation training, children imagined that they were balloons losing all air, or they lay on gymnastic mats stretching their arms and catching various objects (e.g., balloons, scarves, rubber balls). A balancing task comprised activities such as balancing objects on different body parts (e.g., balancing paper cups on the head). Other typical tasks involved playing ballgames in atypical body positions such as walking on heels, tiptoes, or with their hands and feet in parallel. Typical body perception tasks were mastering a path with closed eyes while relying on the instruction of a teammate or breathing with closed eyes and observing their heartbeats.

#### Measures

Phonological awareness was assessed as the dependent variable and measured with the “Test für phonologische Bewusstheitsfähigkeiten” (TPB; Fricke and Schäfer, 2011) that was administered in an individual session. The test consists of an active vocabulary task and seven (out of 11) subtests: (1) segmentation of words into syllables, (2) detection of rhymes, (3) production of rhymes, (4 and 5) synthesis of onset phonemes and words, and (6 and 7) onset phoneme recognition in words. Each subtest consisted of three practice items and 12 test items. The test procedure takes about 40 min and is assessed in individual settings.

The active vocabulary task was necessary to ensure that the children knew the words that were later used in the test because the test items were presented via pictures rather than audio. The children had to name 67 pictures that were used in the subtests. They got one point per picture if they named the picture correctly and without help. The pictures consisted of age-based, daily-vocabulary words with a simple pronunciation and had to be clearly illustratable (e.g., ball, stairs, flower, bottle, table, elephant). This test was not part of any subsequently calculated composite score.

The segmentation of words into syllables subtest 1 consists of picture cards and requires a segmentation of words into syllables by clapping one’s hands (e.g., le-mon, te-le-phone).

In the detection of rhymes task, children were visually presented a paper with four pictures on it. The stimulus was always located on the upper part of the paper, and below were three answer pictures. Children were asked to detect the answer picture that rhymes with the stimulus picture. The possible answer pictures consist of the correct rhyme word, a phonological distracter, and a semantic distracter (e.g., stimulus picture = mouse, correct rhyme picture = house, phonological distractor picture = moose, semantic distractor picture = cheese; this is an invented example in English; the originals were in German).

The production of rhymes subtest 3 consisted of pictures, and the children were told to produce words and non-words that rhyme with the picture (e.g., picture shows a hand, and possible rhymes could be land, sand, fand, band, tand).

The synthesis of onset phonemes and words subtest 4 and 5 required the synthesis of the initial sound and the remaining word into a complete word (e.g., h-and = hand). Here, the words were presented by audio from a CD player and headphones. In subtest 4, the children could choose between three answer pictures; one was the target word, and the other two were phonological and semantic distracters (e.g., target word = hand, phonological distractor = land, semantic distractor = leg). In subtest 5, the children had to reproduce the audio-presented word by saying what they understood.

The onset phoneme recognition tasks required recognition of the initial phoneme in a word. In subtest 6, the same procedure was used as in the detection of rhyme task. Children were asked to detect the picture with the same initial sound as the stimulus picture (e.g., stimulus picture = bus, picture with the same initial sound = bird, phonological distractor = sun, semantic distractor = car). Subtest 7 presents two pictures on a paper with the same initial sound, and the children had to name the sound (e.g., cat and cow = k).

A composite score of all the subtest scores was calculated. The active vocabulary task was not added to the composite score because it only examines whether the children were able to manage the test. In six out of seven subtests, a maximum of 12 points was possible, resulting in a maximum composite score of 72 points. In subtest 3, every correctly produced rhyme word counts as a point. In addition, the following two composite scores were calculated: A phonological awareness score for large phonological units (words), consisting of scores from subtests 1–3, as well as a phonological awareness score for small phonological units (phonemes), consisting of the scores from subtests 4 through 7. Therefore, the maximum score for large phonological units consisted of 24 points plus the points from subtest 3. For small phonological units the maximum score was 48 points.

Control variables such as age, gender, sociodemographic data, intelligence, and children’s enjoyment during the training were measured. The sociodemographic data were assessed from a questionnaire that covered the children’s age and gender. Furthermore, the questionnaire asked for parents’ education and monthly income, and parents’ and children’s music experiences (e.g., playing an instrument, taking private music lessons, singing in a choir). The socioeconomic status was assessed with parents’ education and monthly income. However, only the parents’ educational background could be used for further statistical analyses because nearly half of the parents (44%) did not provide details about their income. For statistical purposes, parents’ education was coded as a dichotomous variable, with 0 for “no university degree,” and 1 for “at least one parent with a university degree.” Parents’ music experience was coded with ‘0’ representing “no music experience,” ‘1’ for “one parent has music experience, e.g., playing an instrument,” and ‘2’ for “both parents have music experience.” The children’s music experience was measured per month.

Furthermore, the language background was assessed with a questionnaire that contained information about the common spoken language in the family’s home environment, where the parents and children were born, and the duration of residence in Germany. The questionnaire was filled out by the parents.

Any kind of kindergarten support program (e.g., preschool program) that was offered by the kindergartens and schools was assessed with a questionnaire filled out by the kindergarten teachers.

To measure intelligence, the culture fair test (CFT1; Weiss and Osterland, 1997), which measures fluid intelligence, was used. The test consisted of five subtests (substitution, mazes, classification, similarities, and matrices) and was administered in groups that did not exceed eight children according to the manual. At least two trained research assistants observed the groups and helped with questions. The duration of test administration was 60 min, including instructions and breaks. Age norms were used to determine the intelligence score for each participant.

Children’s enjoyment during the training was assessed to control for potential biases regarding their willingness and commitment. The measure was a 5-point-smiley scale that was used at the end of every week during the training phase. Children were asked how much they enjoyed the training and were asked to point at one smiley. The meaning of the smileys was explained several times so that the children felt comfortable in their responses. The smiley scale ranged from 1 = “not a bit,” to 5 = “very much.” The smiley in the middle expressed no feelings. For statistical purposes, the smiley points were summed up and divided by the number of training weeks to generate an average score for every child.

### Procedure

Information sheets for the study, informed consent forms, and the sociodemographic questionnaires were handed out to the parents of the preschoolers. The kindergarten teachers had to fill out the questionnaire on whether the participating preschoolers received any kind of additional support programs from the kindergarten and school. The pre-tests started when the parents agreed and had sent back the informed consent as well as the questionnaire. The pre-tests contained an intelligence test and a phonological awareness test.

The intelligence test was performed in groups of four to eight children; phonological awareness was assessed in individual sessions. The assessments were performed on consecutive days, or on 1 day with adequate breaks in between. Subsequent to the pre-tests, 14 weeks of training followed. All training groups (music, phonological skills, and sports) received 20 min of training three times a week. Each group consisted of four to six children. Immediately after the training phase, a post-test was conducted. At the end of the project, each child received a present and a certificate for participation.

## Results

The software SPSS Version 21 was used for statistical analyses. First, the dropout was analyzed to rule out any biases. Children who did not complete more than 70% of the training program were excluded and counted as a dropout. The dropout rate was 37% (23 out of 62 children). The main reason for the high dropout rate was a wave of colds during the wintertime, so that many children were ill and stayed at home several times. A further reason was that many families went on winter holidays so that their participating children were absent for another 1 or 2 weeks. Nevertheless, this dropout rate was not significantly different from the original sample in the control variables of gender, χ^2^ (1, *n* = 57) = 0.869, *p* = 0.351, age, *t*(55) = 0.515, *p* = 0.609, socioeconomic status, *t*(42) = –1.417, *p* = 0.164, and intelligence, *t*(49) = 0.245, *p* = 0.807. Thus, the dropout rate did not bias the sample.

### Control Variables

Differences in gender, age, sociodemographic data (music experience of children and parents, socioeconomic status, language background), and intelligence among the music group, the phonological skills group, and the sport group were compared.

The ratio of males to females was not significantly different between the three groups, χ^2^ (2, *n* = 39) = 0.209, *p* = 0.901; **Table 2**.

**Table 2:** Means and standard deviations for the control variables of age, intelligence (IQ), children’s music experiences, and enjoyment during the training sessions. Also included are the number of males and females in each training group.

Control variable	Music program	Phonological skills program	Sports program
	*M (SD)*	*M (SD)*	*M (SD)*
Age (in months)	72.08 (6.3)	71.55 (6.0)	71.73 (4.2)
Music experiences (in months)	2.38 (7.0)	5.09 (7.6)	0 (0)
IQ	99.67 (12.8)	100.82 (16.4)	94.46 (14.9)
Enjoyment	4.92 (0.2)	4.73 (0.5)	4.87 (0.4)
Gender	7 *m*/6 *f*	5 *m*/6 *f*	7 *m*/8 *f*

Concerning mean age, there were no significant differences between the three groups, *F*(2,36) = 0.029, *p* = 0.971; **Table 2** for means and standard deviations.

With respect to the children’s music experience, there were descriptive differences between the groups, however, regarding the inferential statistics all three groups could be considered as comparable, *F*(2,36) = 2.564, *p* = 0.091, **Table 2**. In addition, the parents’ music experience was not significantly different between the three groups, χ^2^ (6, *n* = 39) = 7.308, *p* = 0.293.

The socioeconomic status did not differ significantly between the three groups, χ^2^ (2, *n* = 30) = 4.826, *p* = 0.090.

The language background was comparable in all three groups. There were no significant differences between the groups in whether one or both parents were born in Germany or abroad, χ^2^ (2, *n* = 37) = 1.401, *p* = 0.496. Furthermore, there were no differences between the groups regarding whether their families spoke more German or their native language at home, χ^2^ (2, *n* = 39) = 3.025, *p* = 0.220.

With respect to intelligence, no significant differences from among the music group, the phonological skills group, and the sports group were revealed, *F*(2,33) = 0.650, *p* = 0.529, **Table 2** for details.

Children’s enjoyment during the training sessions did not differ significantly among the three groups, *F*(2,36) = 0.888, *p* = 0.420, **Table 2**. Thus, the training groups were comparable regarding their enjoyment and willingness to participate.

Taken together, the analyses of the control variables indicated that all groups could be considered as comparable.

### Phonological Awareness

Phonological awareness was assessed prior and after the period of training to establish a specific causal relationship between music training and phonological awareness. Phonological awareness scores were tested with the Kolmogorov-Smirnov (K-S) test, indicating that all variables were normally distributed, all *ps* > 0.178.

The TPB (Fricke and Schäfer, 2011) provides normative data (raw scores and percentile ranks) for every subtest from native speakers of German of the same age groups. All three groups were below average (percentile rank 11–24) for every subtest, except for subtest 1 (segmentation of words into syllables) for the music group and the phonological skills group (both lower than average, percentile rank 25–49).

At the pre-test, prior to the period of training, the three groups did not differ significantly in phonological awareness, *F*(2,33) = 0.588, *p* = 0.561. Furthermore, the composite score for large phonological units revealed no group differences, *F*(2,36) = 0.461, *p* = 0.634, and neither did the composite score for small phonological units, *F*(2,33) = 0.684, *p* = 0.512.

At the post-test, analysis of variances (ANOVAs) with repeated measures were used to find treatment effects in the groups, in a first step for the total score of phonological awareness and in a second and third step for the composite scores for large and small phonological units. In the case of a significant interaction between the groups and the treatments, separate ANOVAs with repeated measures comparing each treatment group (music, phonological skills) to the sports control group were conducted as well as ANOVAs with repeated measures comparing the two treatment groups. Furthermore, *t*-tests specified the differences between the groups. This approach was considered reasonable for analyzing the process of intervention.

**FIGURE 1:**
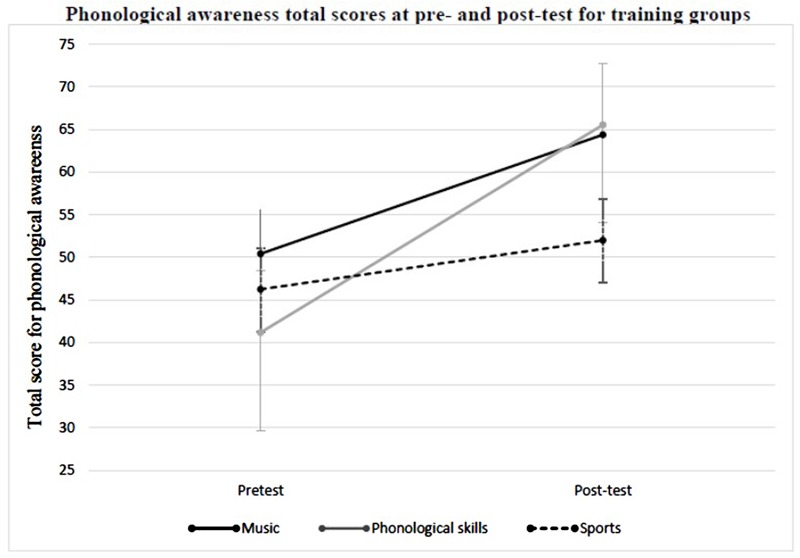
**Mean phonological awareness score for the total score of phonological awareness at pre- and post-test in the music group, the phonological skills group, and the sports group**.

In a first step, the total score for phonological awareness was entered in a 3 (group: music, phonological skills, sports) × 2 (time: pre-test vs. post-test) ANOVA with repeated measures on the last factor. This analysis revealed a significant main effect of time, *F*(1,33) = 51.361, *p* < 0.001, η^*2*^ = 0.609, with phonological awareness improving significantly from pre-test to post-test. No significant main effect for group was revealed, *F*(2,33) = 0.286, *p* = 0.753, η^*2*^ = 0.017. Furthermore, the analysis revealed a significant group × time interaction, *F*(2,33) = 4.074, *p* = 0.026, η^*2*^ = 0.198; **Figure 1**.

For the comparison between the music group and the sports control group, the total score of phonological awareness was entered in a 2 (group: music, sports) × 2 (time: pre-test vs. post-test) ANOVA with repeated measures on the last factor. This analysis revealed a significant main effect of time, *F*(1,23) = 25.541, *p* < 0.001, η^*2*^ = 0.526. Phonological awareness improved significantly from pre-test to post-test. No significant main effect for group was revealed, *F*(1,23) = 0.879, *p* = 0.358, η^*2*^ = 0.037. Furthermore, the analysis showed no significant group × time interaction, *F*(1,23) = 1.825, *p* = 0.190, η^*2*^ = 0.074.

For the comparison between the phonological skills group and the sports control group, the total score of phonological awareness was entered into a 2 (group: phonological skills, sports) × 2 (time: pre-test vs. post-test) ANOVA with repeated measures on the last factor. This analysis revealed a significant main effect of time, *F*(1,21) = 35.079, *p* < 0.001, η^*2*^ = 0.626. Phonological awareness improved significantly from pre-test to post-test. There was no significant main effect for group, *F*(1,21) = 0.079, *p* = 0.781, η^*2*^ = 0.004. Additionally, this analysis revealed a significant group × time interaction, *F*(1,21) = 8.047, *p* = 0.010, η^*2*^ = 0.277. Subsequent independent *t*-tests showed that the phonological skills group and the sports control group differed significantly at neither the pre-test, *t*(22) = –0.495, *p* = 0.627, nor at the post-test, *t*(24) = 1.120, *p* = 0.282. Comparing the pre-test and post-test measures of the total score of phonological awareness within each group with a dependent *t*-test, the phonological skills group showed a significant increase, *t*(10) = –4.865, *p* = 0.001, as well as the sports control group, *t*(11) = –3.165, *p* = 0.009. However, the increase in the phonological skills group is much higher than in the sports control group, as the significant interaction demonstrates (**Figure 1**).

For the comparison of the two treatment groups, the total score of phonological awareness was entered into a 2 (group: music, phonological skills) × 2 (time: pre-test vs. post-test) ANOVA with repeated measures on the last factor. This analysis showed a significant main effect of time, *F*(1,22) = 41.363, *p* < 0.001, η^*2*^ = 0.653, with phonological awareness improving significantly from pre-test to post-test. No significant main effect for group was revealed, *F*(1,22) = 0.149, *p* = 0.703, η^*2*^ = 0.007. Furthermore, this analysis showed no significant group × time interaction, *F*(1,22) = 2.437, *p* = 0.133, η^*2*^ = 0.100.

Taken together, after the period of training, the phonological skills group showed a significant increase in the total score for phonological awareness compared to the sports control group. However, the subsequent independent *t*-tests showed no significant differences between the groups at the post-test, possibly due to large variance within the phonological skills group (*M* = 65.546, *SD* = 36.634). None of the other group comparisons revealed significant differences.

**FIGURE 2:**
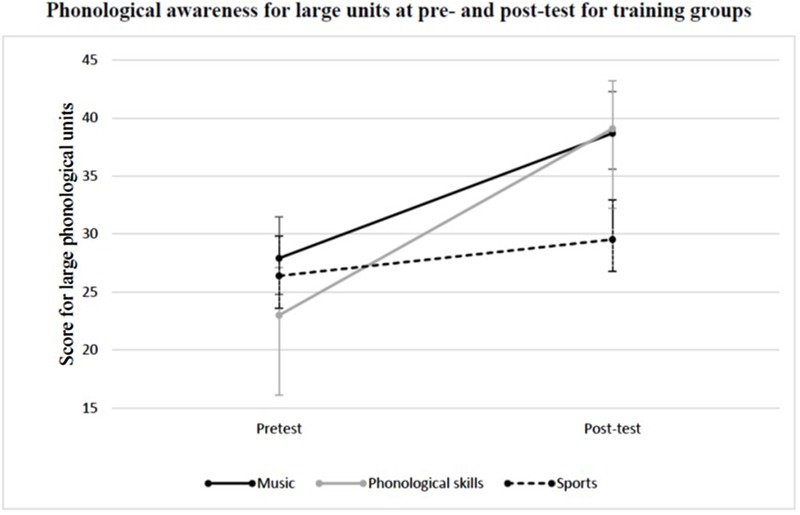
**Mean phonological awareness score for large phonological units at pre- and post-test in the music group, the phonological skills group, and the sports control group**.

In a second step, the groups’ phonological awareness on the word level (larger phonological units) was compared. Therefore, the composite score for large phonological units was entered into a 3 (group: music, phonological skills, sports) × 2 (time: pre-test vs. post-test) ANOVA with repeated measures on the last factor. This analysis revealed a significant main effect of time, *F*(1,36) = 37.847, *p* < 0.001, η^*2*^ = 0.513. Phonological awareness improved significantly from pre-test to post-test. No significant main effect for group was found, *F*(2,36) = 0.561, *p* = 0.576, η^*2*^ = 0.030. Furthermore, this analysis revealed a significant group × time interaction, *F*(2,36) = 5.483, *p* = 0.008, η^*2*^ = 0.233; **Figure 2**.

For the comparison between the music group and the sports control group (2 × 2 ANOVA with repeated measures on the last factor), the analysis revealed a significant main effect of time, *F*(1,26), *p* < 0.001, η^*2*^ = 0.395. Phonological awareness improved significantly from pre-test to post-test. No significant main effect for group was found, *F*(1,26) = 1.663, *p* = 0.209, η^*2*^ = 0.060. Furthermore, this analysis showed a significant group × time interaction, *F*(1,26) = 5.021, *p* = 0.034, η^*2*^ = 0.162. The effect size was *dcorr* = 0.82. Subsequent independent *t*-tests showed that the music group and the sports control group did not differ significantly at the pre-test, *t*(27) = 0.470, *p* = 0.642, but a significant difference was revealed at the post-test, *t*(27) = 2.278, *p* = 0.031. Dependent *t*-tests demonstrated a significant increase in the phonological awareness on the word level in the music group, *t*(12) = –4.268, *p* = 0.001, whereas no such improvement was found for the sports control group, *t*(14) = –1.399, *p* = 0.184.

For the comparison between the phonological skills group and the sports control group (2 × 2 ANOVA with repeated measures on the last factor), this analysis revealed a significant main effect of time, *F*(1,24) = 21.268, *p* < 0.001, η^*2*^ = 0.470, with phonological awareness improving significantly from pre-test to post-test. No significant main effect for group was revealed, *F*(1,24) = 0.296, *p* = 0.592, η^*2*^ = 0.012. Additionally, this analysis showed a significant group × time interaction, *F*(1,24) = 9.662, *p* = 0.005, η^*2*^ = 0.287. The effect size was *dcorr* = 0.35. Subsequent independent *t*-tests showed that the phonological skills group and the sports control group differed significantly at neither the pre-test, *t*(25) = –0.517, *p* = 0.610, nor at the post-test, *t*(24) = 1.291, *p* = 0.219. Comparing the pre-test and post-test measure of the composite score for large phonological units within each group with a dependent *t*-test, the phonological skills group showed a significant increase, *t*(10) = –4.228, *p* = 0.002. As stated earlier, the sports control group did not show such an improvement, *t*(14) = –1.399, *p* = 0.184.

For the comparison between the music group and the phonological skills group (2 × 2 ANOVA with repeated measures on the last factor), this analysis showed a significant main effect of time, *F*(1,22) = 36.549, *p* < 0.001, η^*2*^ = 0.624, with phonological awareness improving significantly from pre-test to post-test. No significant main effect of group was revealed, *F*(1,22) = 0.129, *p* = 0.723, η^*2*^ = 0.006. Furthermore, the analysis showed no significant group × time interaction, *F*(1,22) = 1.536, *p* = 0.228, η^*2*^ = 0.065.

Hence, after the period of training, both treatment groups showed a significant increase in the composite score for large phonological units compared to the sports control group. The comparison of the two treatment groups did not reveal a significant difference. The fact that the independent *t*-test between the phonological skills groups and the sports control group revealed no significant differences at the post-test could be explained by large variance within the phonological skills group (*M* = 39.091, *SD* = 22.762).

**FIGURE 3:**
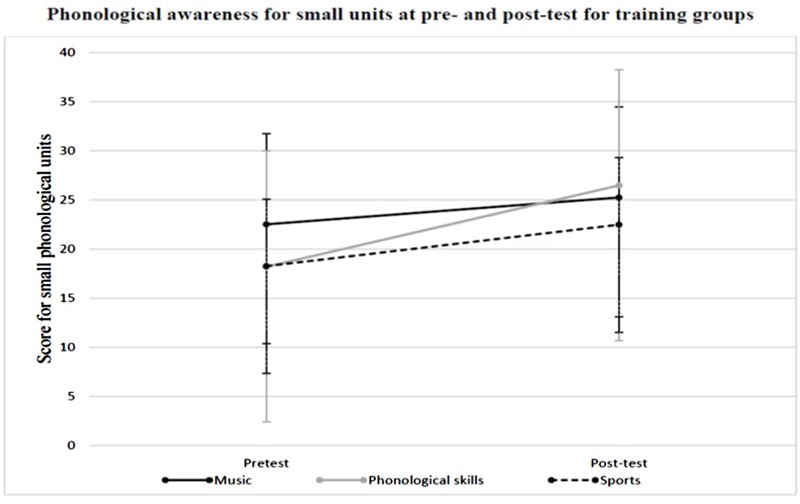
**Mean phonological awareness score for small phonological units at pre- and post-test in the music group, the phonological skills group, and the sport control group**.

In a third step, the groups’ phonological awareness on the phoneme level (small phonological units) was compared. Thus, the composite score for small phonological units was entered into a 3 (group: music, phonological skills, sports) × 2 (time: pre-test vs. post-test) ANOVA with repeated measures on the last factor. This analysis revealed a significant main effect of time, *F*(1,33) = 21.267, *p* = < 0.001, η^*2*^ = 0.392 with phonological awareness improving significantly from pre-test to post-test. No significant main effect of group was found, *F*(2,33) = 0.220, *p* = 0.804, η^*2*^ = 0.013. Furthermore, no significant group × time interaction was revealed, *F*(2,33) = 0.741, *p* = 0.484, η^*2*^ = 0.043, **Figure 3**. In light of these findings, no further statistical analysis for the phonological awareness on the phoneme level was conducted.

## Discussion

The aim of this study was to investigate the effect of a music program on phonological awareness in preschoolers from immigrant families. This effect was compared to the effect of a phonological skills program that directly addresses phonological awareness, and a sports program from which no effect on phonological awareness was expected. Accordingly, preschoolers from immigrant families were randomly assigned to a music program, a phonological skills program, or a sports program that served as the control group. Children were trained in small groups, three times a week for 20 min per session, over a period of 14 weeks. Before and after the training period, participants were individually tested on phonological awareness.

At the pre-test, no significant differences were found between the three groups regarding the control variables (age, gender, socioeconomic status, children’s and parents’ music experiences, language background, country of birth, kindergarten support programs, and intelligence). Furthermore, no differences in phonological awareness (total score, large phonological units, small phonological units) were revealed at the pre-test. Randomization yielded comparable groups.

At the post-test, a positive effect of the music program and the phonological skills program was revealed in phonological awareness of large phonological units. Both treatment groups improved significantly in phonological awareness of large phonological units, in contrast to the sports control group, which showed only a slight increase. In addition, the phonological skills group showed a significant increase in the total score of phonological awareness, which comprises large and small phonological units, compared to the sports control group. All three groups showed a similar development in phonological awareness of small phonological units, indicating an overall effect of maturation. Thus, this study provides evidence that the music program as well as the phonological skills program promote phonological awareness in preschoolers of immigrant families, particularly phonological awareness of large phonological units. More than half of the children reported speaking no German or only little German at home with their families. Accordingly, the rare opportunity to practice the second language in the home environment and the missing language model for the grammatical structure (Tracy, 2009) were possible reasons for the poor German language level that many children had. Also, phonological awareness scores at pre-test were mostly below average compared to normative data from the TPB (Fricke and Schäfer, 2011), and confirm the phonological deficits of children of immigrants (Triarchi-Herrmann, 2009). Despite language barriers and different ethnical backgrounds, the music training program conveyed extensive competencies. The participants were familiarized with more than 30 songs for children, they learned the names of different percussion instruments, and they learned how to play them. Additionally and unconsciously, the music training program increased their ability of phonological awareness in a second language. Thus, the current result is remarkable because such a heterogeneous group of preschoolers profited from a comprehensive music training program in a particular and important ability for schooling, namely phonological awareness.

This result is consistent with previous findings of Lamb and Gregory (1993) as well as Anvari et al. (2002). Their correlational studies revealed an association between musical abilities and phonological awareness in 4- and 5-year-old children, although their participants did not come from immigrant families.

Our results are in line with the few studies that have also investigated the effect of music programs on phonological awareness in children not from immigrant families (Gromko, 2005; Bolduc, 2009; Degé and Schwarzer, 2011; Herrera et al., 2011; François et al., 2013; Moritz et al., 2013). Moritz et al. (2013) as well as Bolduc (2009) demonstrated positive effects of music on phonological awareness by investigating different music programs. However, both studies lacked a control group without music, so the general role of music training remained unclear. Therefore, the present study emphasizes the importance of a control group. Due to the sports control group receiving the same amount of training time as the experimental groups, any effects of extra attention were excluded. Furthermore, it is possible to infer the degree of increase in phonological awareness between the treatment (music, phonological skills) and control (sports) programs. Gromko (2005) revealed a significant increase in phonological awareness by training kindergarten children with a music program for 4 months. However, this study was based on a pseudo-random assignment of the children to the treatment or the no-treatment control group. This meant that there was no control for systematic differences between the groups, which in turn limited the comparability of the groups. In addition, it is possible that the treatment group represented an effect of extra attention compared to the no-treatment control group. In contrast to Gromko’s (2005) study, our investigation used an experimental design with randomized assignment of the participants to a treatment or control group. Thus, systematic differences between the groups were ruled out. Due to this approach, it was possible to establish a specific causal relationship between music training and phonological awareness, especially on the word level. The study from Herrera et al. (2011), used phonological training with music that was designed for conveying phonological content. In contrast, our study used a comprehensive music training program that is primarily appropriate for early music education. Following our results, a conventional music training program that is not explicitly designed for promoting phonological skills, still enhances phonological awareness in preschoolers of immigrants.

The results of this study match with the OPERA hypothesis from Patel (2011) because the music program reflects all conditions: (1) the music training could tap into shared neural resources of music and speech (Koelsch and Siebel, 2005; Schön et al., 2010; Patel, 2011, 2014) that guarantees the overlap, (2) rhythm exercises, joint drumming and singing required high precision during the music training sessions, (3) the music training consisted basically of joint musical activities that offered emotional rewards, (4) all exercises (e.g., learning songs, meter execution tasks, joint drumming) were often repeated, and (5) small groups of four to six children ensured a highly attentive training atmosphere. Furthermore, the revealed effect is in line with the hypothesis of Besson et al. (2011) that focuses on frequency and duration as important auditory parameters for both music and speech. Also, the PATH postulated by Tierney and Kraus (2014) confirm the result of the large effect size. Music training promotes timing precision. This higher precision also benefits speech sound perception, which is important for phonological awareness. Nevertheless, there is no evidence in this study that music training enhances phonological awareness of small phonological units. Thus, this result is not consistent with Patel’s hypotheses (Patel, 2008). This model especially predicts effects on the phoneme level because of the postulated categorical building blocks of notes and phonemes that are related to each other. Furthermore, the mentioned hypotheses from Besson et al. (2011) and Tierney and Kraus (2014) refer to the phonological awareness of large and small phonological units. Accordingly, important auditory parameters, such as frequency and duration (Besson et al., 2011) or timing precision (Tierney and Kraus, 2014) should also affect phonological awareness on the phoneme level. Possibly, the training period of 14 weeks was too short to affect the auditory sensitivity on such an absolutely precise level that is important for the extraction and manipulation of phonemes. In contrast, Moritz et al. (2013) had a training period of 1 year, Gromko (2005) had a four-month-training period, and Herrera et al. (2011) had at least 16 weeks of training.

This study replicated the results of Degé and Schwarzer (2011), who revealed a positive effect of a music program on phonological awareness in preschoolers at the same age but not from immigrant families. In the study of Degé and Schwarzer (2011) as well as in this experiment, it was shown that phonological awareness of large phonological units was more intensely affected by both the music and phonological skills programs relative to small phonological units. The replication of their data provides evidence that the music program has a larger effect size compared to the phonological skills program. The music program particularly affects large phonological units by practicing rhythmical exercises and singing songs with catchy rhythms and rhymes (Degé and Schwarzer, 2011).

Concerning small phonological units, the effects of maturation and preschool activities in the kindergartens (Marx, 2007) could be possible explanations for the similar development across the three groups. However, the phonological skills program addressed small phonological units so that an effect might have been expected (Degé and Schwarzer, 2011).

Although we used a similar design as the Degé and Schwarzer (2011) study, some important differences did exist. This study reduced the training phase from 20 weeks to 14 weeks. Furthermore, the frequency of training was changed from 10 min every day to three times a week for 20 min each. These reductions made it possible and convenient for the kindergartens to include the study program in their schedule. Hence, our study also showed that it is possible to train phonological awareness with music in a non-daily routine. In addition, we revealed that it is possible to elongate learning periods for the preschoolers to 20 min without losing an effect on learning outcome. Despite these methodological modifications compared to Degé and Schwarzer (2011), the results clearly indicate the positive effect size of a music program on phonological awareness of large phonological units in preschoolers from immigrant families.

So far, no cited study assessed motivational aspects of the children’s participation in a program. By contrast, this experiment explicitly assessed the children’s enjoyment during the training phase. Although this variable did not differ significantly among the three groups, the children in the music program showed the highest mean level of enjoyment compared to the other groups (**Table 2**). It is obvious that the children enjoyed the programs equally, because all participants showed high mean scores, and all programs were designed explicitly for preschoolers. However, it might be possible that the children in the music group enjoyed the training more because musical activity is known to induce positive emotions as well as to inspire and motivate (Hesse, 2003). Children of immigrant families may especially benefit from the inspiring and motivating aspect of music that facilitates learning processes (Wolf et al., 2000). The large effect size of the music program (*dcorr* = 0.82) on phonological awareness of large phonological units compared to the phonological skills program (*dcorr* = 0.35) is especially notable. The music program only addresses phonological awareness indirectly, whereas the phonological skills program is explicitly designed to train phonological awareness. These effect sizes are slightly smaller compared to the effect sizes found in the study from Degé and Schwarzer (2011).

### Limitations and Future Directions

A limitation of this study is the lack of control mechanisms during the training phase. It remains unclear whether children used any kind of language support during the training phase, such as the parents practicing phonological awareness with the children at home or that children started a language course apart from the kindergarten. In this experiment, a questionnaire addressed these issues only prior to the start of the training. Future research should control these influences by sending questionnaires to the parents at given dates during the training phase.

Unfortunately, this experiment had no follow-up measure, which would be desirable to analyze the sustainability of the effects. However, participating children in this study were between 4 and 6 years old. A follow-up measure, perhaps 1 year later, would mean that at least some of these children would go to school, and schooling causes a high increase in phonological awareness (Marx, 2007). The comparability between the groups could not be guaranteed anymore, except that the school children would be eliminated; that, in turn, diminishes the sample size.

This study consisted of only a small sample size in the treatment and control groups. One reason for this was due to difficulties in the recruitment of the participants due to language barriers. Another reason was due to the large number of absent days of many of the children due to illness and holidays, such that they could not attend the whole training or at least 70% of the training sessions.

Despite the small sample size in this study, we found a large effect size of music training on phonological awareness, especially, on the word level comprising blending, segmentation, and rhyming. The result of this study indicates a specific causation between music training and the promotion of phonological awareness in children of immigrants. Furthermore, this study supports the results of Degé and Schwarzer (2011) and extends their findings to this special target group. Thus, a comprehensive music program could be used as an additional opportunity to promote phonological skills in children of immigrant families.

## Author Contributions

All authors developed the concept. HP conducted the study and wrote the first draft of the manuscript. All authors contributed to and have approved the final manuscript.

## Conflict of Interest Statement

The authors declare that the research was conducted in the absence of any commercial or financial relationships that could be construed as a potential conflict of interest.
